# Utilising datasheets for the informed automated design and build of a synthetic metabolic pathway

**DOI:** 10.1186/s13036-019-0141-z

**Published:** 2019-01-18

**Authors:** Kealan Exley, Christopher Robert Reynolds, Lorna Suckling, Soo Mei Chee, Argyro Tsipa, Paul S. Freemont, David McClymont, Richard Ian Kitney

**Affiliations:** 1 Department of Bioengineering, Imperial College London, London, UK; 2 Imperial College Centre for Synthetic Biology, Imperial College London, London, UK; 3 The London DNA Foundry, Imperial College London, London, UK; 4 SynbiCITE, Imperial College London, London, UK; 5 Section of Structural Biology, Department of Medicine, Imperial College London, London, UK

**Keywords:** Synthetic biology, Datasheets, Design of Experiment (DoE), Automation workflow

## Abstract

**Background:**

The automation of modular cloning methodologies permits the assembly of many genetic designs. Utilising characterised biological parts aids in the design and redesign of genetic pathways. The characterisation information held on datasheets can be used to determine whether a biological part meets the design requirements. To manage the design of genetic pathways, researchers have turned to modelling-based computer aided design software tools.

**Result:**

An automated workflow has been developed for the design and build of heterologous metabolic pathways. In addition, to demonstrate the powers of electronic datasheets we have developed software which can transfer part information from a datasheet to the Design of Experiment software JMP. To this end we were able to use Design of Experiment software to rationally design and test randomised samples from the design space of a lycopene pathway in *E. coli*. This pathway was optimised by individually modulating the promoter strength, RBS strength, and gene order targets.

**Conclusion:**

The use of standardised and characterised biological parts will empower a design-oriented synthetic biology for the forward engineering of heterologous expression systems. A Design of Experiment approach streamlines the design-build-test cycle to achieve optimised solutions in biodesign. Developed automated workflows provide effective transfer of information between characterised information (in the form of datasheets) and DoE software.

**Electronic supplementary material:**

The online version of this article (10.1186/s13036-019-0141-z) contains supplementary material, which is available to authorized users.

## Background

One of the principal ideas behind synthetic biology is the utilisation of standardised biological parts for the assembly of genetic devices, circuits and pathways [1, 2]. As the field has developed, so too has the availability of standard parts; hastened by the arrival of cloning toolkits for DNA assembly [3, 4]. These toolkits are often designed for one-pot restriction-ligation based cloning of distinct parts, with many of the parts associated with characterisation data [4]. Although the number of standardised parts has increased, the representation of the part information and characterisation data has largely remained non-standardised [5]. Often, the characterisation information for a given part is retained in the rigid format of a research paper. Whereas, it is critical that a consistent standardised reporting system exists to augment the sharing of characterisation data and information relating to a part or biological component [6, 7].

Datasheets have been proposed as one method to enable the standardisation of part information and data [8–10]. Datasheets, which are widely used in engineering, contain quantitative information to aid in model design and the identification of experimental parameters [1]. The first basic datasheets for synthetic biology were described in 2008 to represent data from a cell-to-cell communication receiver part, BBa_F2620 [9]. The datasheet comprised graphical, tabular and written information on the biological part, presenting a mix of qualitative information (structure description, summary of its function) and quantitative measurements (inducer dose response and fluorescent output from the device) [9].

To date, however, there has been limited uptake of datasheets by synthetic biologists, possibly because the focus has been on human-readable datasheets and their content [3]. This has made it difficult to quickly disseminate part information. Recently, electronic datasheets have been developed, that are both ‘human-readable’ and structured and formatted in such a way that software can easily retrieve and process the data and information making them also ‘machine readable’ [11]. This enables the serialisation of the electronic datasheets to an Synthetic Biology Open Language (SBOL) standard [11]. SBOL is an emerging standard in synthetic biology for the description and exchange of biological designs. The SBOL standard captures a ‘common core’ of the basic features of a biological part, representing its biological structure, function, and sequence [12]. The standard can be used to extract relevant information for downstream processing software and data models. For instance, design and mathematical models can be constructed using the SBOL standard with computer aided design (CAD) tools such as iBioSim [13], and TinkerCell [14]. The development of electronic datasheets enables the dissemination of part characterisation information into these and other design software tools.

Design software tools are becoming essential with increased automation of modular multi-part DNA assembly methods. These methods enable the high-throughput building of hundreds if not thousands of designs [15–17]. Design of Experiment (DoE) software is particularly useful in determining an optimal design. The alternative is the sequential analysis of each build from the design space; this approach would be very time consuming and costly. Instead, it is more beneficial to use a DoE approach to examine a randomised multifactorial design space [18–20]. DoE has been extensively used in bioprocess engineering to optimise downstream and upstream processes, such as bioreactor growth conditions and protein purification [21]. Recently, statistical model-based DoE procedures have been applied to genetic pathway design and to define automation methodology for part assembly [20, 22, 23]. DoE software, such as the JMP custom design tool, enables a defined set of controlled experiments to be conducted in randomised order to prevent biases. For genetic pathway design, the experimental variables are parts, with the part information utilised for the coordinated design and construction of a genetic library.

In this paper, in order to demonstrate the applicability of electronic datasheets for metabolic genetic pathway design, part information from a set of standard biological parts was uploaded to DoE software, JMP. This was used in the pathway design and analysis of a built heterologous metabolic pathway. We chose to investigate the production of the carotenoid, lycopene in *E. coli*. Carotenoids, such as lycopene, have long been used as food colorants due to their pigmentation. Carotenoids have also demonstrated to have potential as nutraceuticals and pharmaceuticals [24]. For instance, the antioxidant properties of lycopene have demonstrated to have anticancer properties against prostate cancer [25, 26]. More recently, the synthesis of lycopene and other carotenoids have become model metabolic pathway in synthetic biology. This is because the colour pigmentation of cells, which enables a colorimetric detection of successful synthetic pathways.

In-house software has been developed for this paper which retrieves part information of the biological parts from an online data repository of datasheets. The in-house software ranks biological parts such as RBSs and promoters, which are classified as discrete numeric factors by JMP design tool. The ranking of biological parts will enhance the modelling of metabolic pathways by linking the relative strengths of parts to titre levels. By utilising DoE approach, with the ranking of biological parts, it is possible to determine, on the basis of design principles, which genetic configuration will lead to improvements of cellular properties and product yields.

## Results

### Implementing an automated design framework

**Fig. 1:**
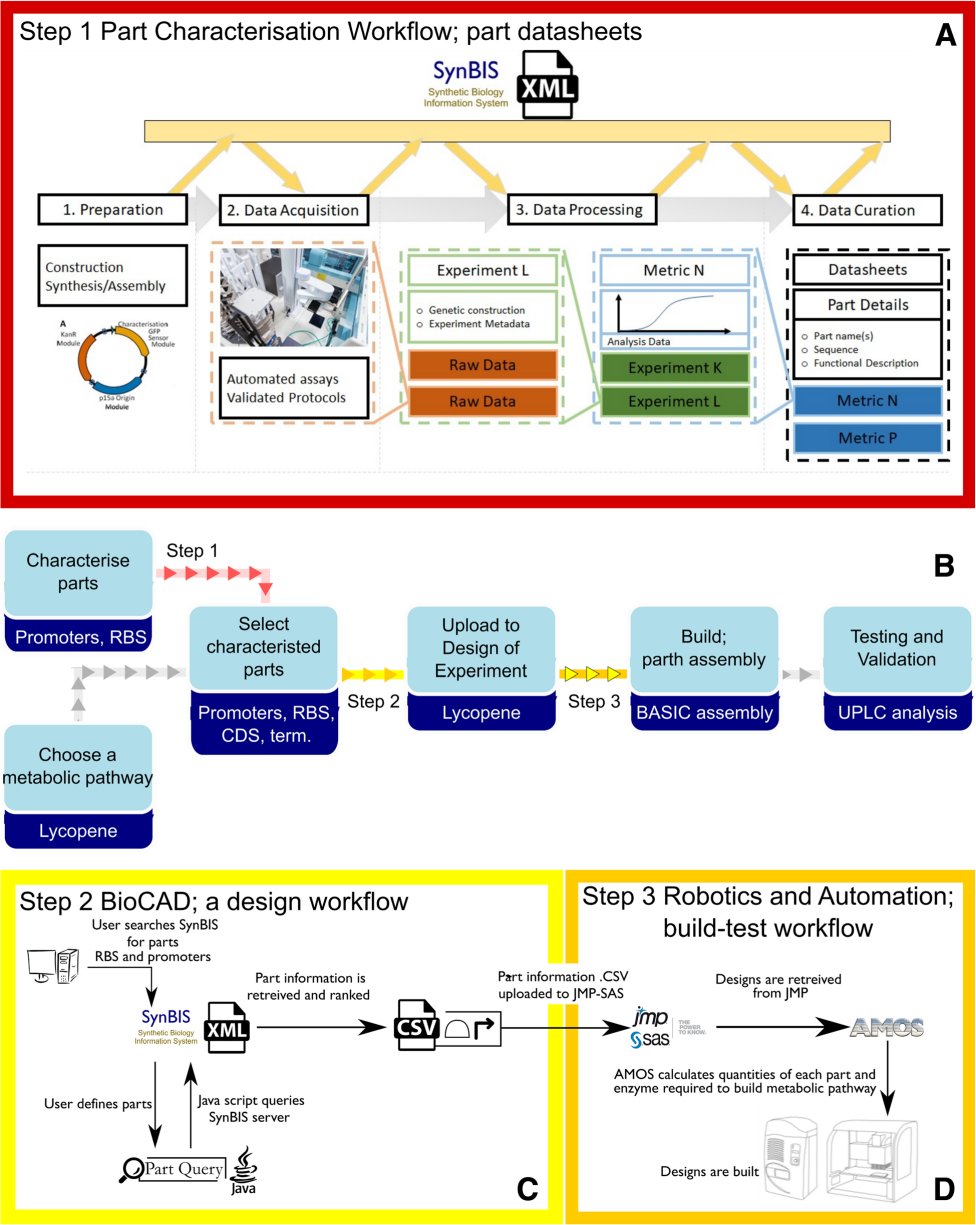
Diagram of the automated workflow for the designing and building of heterologous metabolic pathways; To aid in the development of new novel metabolic pathways in microbial hosts a design-build framework was established. To build a metabolic pathway, information from data repositories is fed into BioCAD software. BioCAD constructs a comprehensive virtual assembly of every potential pathway design. Once designed, metabolic pathways are constructed and tested using automated liquid handling robotics. **b** To achieve an automated design-build framework with minimal human involvement, in-house software tools and data models have been developed. These tools have been installed into automated pipeline, represented by the red or yellow arrowed lines. The grey and coloured arrowed lines represent the flow of information through the process development of a heterologous metabolic pathway. **a** A workflow and data model has been developed to capture and disseminate part information to an online datasheet repository known as SynBIS. **c** An in-house software tool has been developed to convert SBOL serialised datasheets to readable CSV files to input biological part information into the JMP DoE software tool. Screenshots of the software and CSV file can be seen in the supplementary information (Additional file 1: Figure S1). **d** JMP DoE software tool can be utilised to design biosynthetic genetic pathways. The software tool AMOS has been developed to coordinate the building of selected samples from the design space created by JMP in liquid handling robots

An automated design-build framework has been developed to streamline information flow between individual steps in a rapid prototyping pipeline (Fig. 1b). This ensures accuracy and standardisation in the design and build of heterologous biosynthetic pathways. The pipeline developed minimises human involvement by; (Step 1) linking data produced from characterisation experiments to datasheets (Fig. 1a), (Step 2) linking the data from datasheets to design tools (for the design and model of new biosynthetic pathways) (Fig. 1c), and (Step 3) linking genetic configurations from the design space to liquid-handling robotics for the assembly of intended genetic metabolic pathways (Fig. 1d). The three different steps of the automated design and build pipeline are represented in Fig. 1. The first and final steps have previously been described and implemented within the London DNA foundry at Imperial College (see references [11] and [22]). However, to achieve a complete design-build framework, a link is required between characterised data of biological parts essential to build genetic pathways and the tools used to design these pathways, such as the DoE software tool JMP. To provide this, in-house software was developed, which utilises the detailed information found on electronic datasheets.

As shown in Fig. 1a, the first step in the systemic design workflow utilises a data model for generation of readable datasheets from raw data acquired from a standardised automated protocol. The raw data is achieved through the in vivo monitoring of GFP production from a uniformed cell growth rate. The cell growth is maintained through a dilution and sub-culturing protocol using liquid-handling robotics within constant environmental conditions (e.g. *media* and temperature) [27]. This ensures that when repeating the characterisation protocol, a consistent replication of cell population dynamics is achieved each time. Once raw data is acquired, a data model then interprets the raw data with the inclusion of data calibrations to relatively quantify the part. For instance, constitutive promoters are measured against the reference constitutive promoter J23101 as following the methodology set out by Kelly et al. [28]. Once characterisation data is collected on a part, the data model then disseminates the biologically-relevant metrics and other part information on datasheets. The datasheets include three main categories 1) sequence description, 2) the results of the data model, demonstrating the relative quantification of the part (e.g. Relative Promoter Unit of a constitutive promoter, and 3) Raw data acquisition (e.g. plate reader or flow cytometry information). The datasheets are formalised in a manner which enables their serialisation to synthetic biology standards such as DICOM-SB and SBOL [11, 29]. The datasheets conveying the part information are accessible on the web-based biological part repository SynBIS (http://synbis.bg.ic.ac.uk). Currently, the behavioural quantification or characterisation of a parts is limited to promoters and RBSs (i.e. parts related to protein production). An example of a datasheet can be found in the supplementary information of this paper (Additional file 1: Figure S4) and further details on the data model can be found in references [11, 29].

To link the datasheets to DoE software, a Java-based application was developed to retrieve part information from the SynBIS XML framework. The application provides the user with a graphical interface that allows the specification of a series of part IDs (Additional file 1: Figure S1). The programme queries the SynBIS server to retrieve XML data - specifying, for each part; the native host, DNA sequence and relative strength of the part. In addition, to aid in the design and modelling the biological parts are ranked, with the rank order determined by the relative strength of the utilised parts (with ‘1’ being the lowest strength). The ranked ordering of biological part data can then be exported as a comma-separated variable (CSV) file, as the machine-readable delimited text is suitable for loading to DoE design software tool JMP.

The JMP custom design tool is valuable in the implementation of an automated design-build framework. It enables integrated modelling and analysis of experimental variables, to determine statistically relevant parameters which can be utilised to inform design. In this case, the experimental variables are biological parts represented as discrete numeric factors. The JMP software constructs full factorial designs for any number of assigned continuous, discrete numeric or categorical factors. To aid in the design, the discrete numeric factors are ranked by the developed in-house software [30]. Therefore, to aid in returning statistical relevant models, the parameters (i.e. parts) should be accurately characterised.

### Design and construction of lycopene operon

**Fig. 2:**
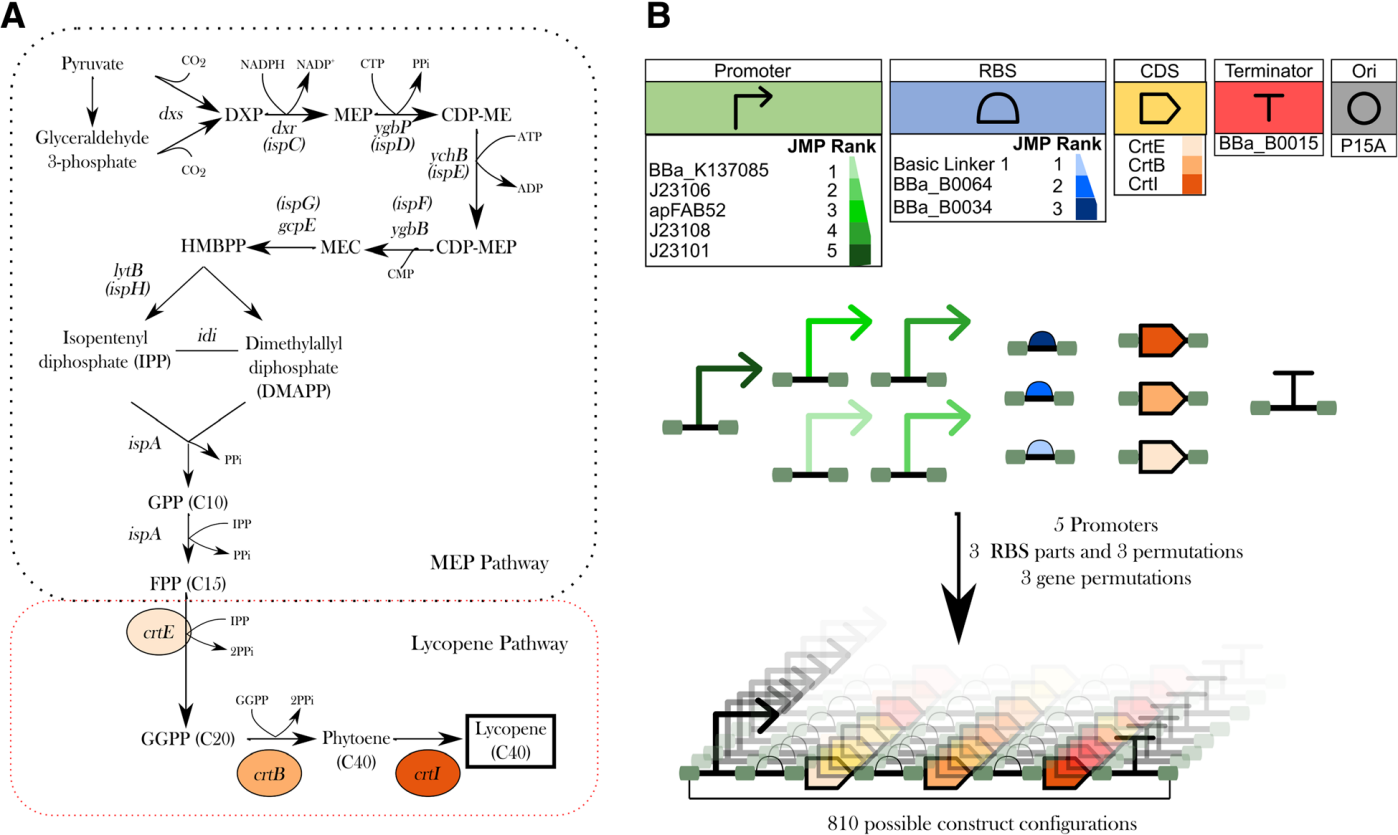
The biological parts utilised for combinatorial construction of a lycopene biosynthetic pathway. **a** The carotenoid lycopene is produced in *E. coli* through a heterologous biosynthetic pathway composed of three enzymes (CrtE. CrtB and CrtI). The heterologous proteins use FPP, which is a product of MEP pathway, as a precursor. **b** A complete combinatorial library totalling 810 pathway configurations can be designed by varying the promoter and RBS parts and by varying the order of pathway genes and RBS parts. Through the application of statistical modelling by DoE software, the designed library was reduced to 88 representative constructs, A table displaying part information of each construct is displayed in the supplementary information ( Additional file 1: Table S1). The pathway library was assembled and expressed in *E. coli* DH5α to test lycopene titres. The relative effects of the different design factors had on the titres level of lycopene were modelled using JMP. The model identified biological parts which effected titre levels, this can help aid future designing of the lycopene biosynthetic pathway.

To produce the carotenoid lycopene in *E. coli,* a synthetic operon was designed to coordinate and regulate the transcription and translation of the three exogenous enzymes (Fig. 2). The red-coloured lycopene is produced through the conversion of isopentenyl pyrophosphate (IPP) and farnesyl pyrophosphate (FPP) by three exogenous enzymes GGPP synthase (crtE), phytoene synthase (crtB), and phytoene desaturase (crtI) [31]. The pyrophosphates IPP and FPP act as precursors in the non-mevalonate pathway (MEP pathway) in *E. coli* [32]*.*

The different parts which constitute the lycopene biosynthesis operon were selected from either the SynBIS repository or the iGEM Registry of Standard Biological Parts [33]. The operon was constructed from one of five different constitutive promoters. The five constitutive promoters were selected based on a low to medium RPU (relative promoter units). Previous reports on the production of lycopene had suggested that exogenous protein levels should be low in order to not divert vital metabolic flux away from the essential pathways which utilise IPP and/or FPP [32]. A total of three RBS parts were selected, which covered a range of strengths (low, medium and high). In addition, the operon contained the three CDS parts of the lycopene pathway and a terminator (Fig. 2). These were placed into a low-copy number plasmid with a p15A origin of replication (Ori). This multivariate approach of the 14 distinct parts, with varying promoters, RBS and, 18 permutations of RBS and the gene location. This resulted in full factorial design of 810 possible configurations of the lycopene operon ((5*3*3*1*1)*18).

The experimental factors and response variables were inputted into the JMP custom design tool using the CSV converter software. For the purposes of modelling, biological parts of RBS in each position and promoters are considered as discrete numeric factors, while gene order is considered as a categorical factor with six possible values. The discrete numeric factors are uploaded as numbers, which indicate the rank order of the part based on the strength. Once added, JMP generates a reduced random set of experimental conditions from the full factorial designs. For this study, a reduced set of 88 design formats were calculated, achieving a compression ratio of 46:5. The 88 design assemblies based on samples from the design space are described in the supplementary information (Additional file 1: Table S1).

The synthetic operon was assembled using the BASIC modular cloning method [34]. Consequently, all part classes (excluding the RBS) were synthesised with prefix and suffix sequences to enable BASIC orthogonal linker-based DNA assembly. The RBS parts were included within linker sequences required to assemble the operon. The BASIC cloning reaction, similar to other modular cloning toolkits, has been automated to enable robust and high-throughput assemblies at nanolitre scale [35]. The custom LIMS software tool, AMOS, was used to coordinate the assembly of the 88 selected lycopene operon variants. This was achieved by managing the combination of parts, linkers, enzymes and buffers across multiple reactions using a liquid handler [22]. The in-house software AMOS also assigns each design an ID (also known as stitch ID) to each construct.

### Testing and characterisation of the lycopene operon variants

An initial characterisation was performed to obtain an overview of lycopene concentration from a randomised set of 88 constructs of the lycopene pathway. These results were utilised to identify optimal part combinations. The results demonstrated that of the 88 assembled samples from the lycopene pathway design space 53% failed to produce lycopene, with 45 colonies exhibiting no observable growth and 2 colonies exhibiting growth but with no detectable lycopene (Additional file 1: Figure S2). The results indicated a trend, i.e. as the relative strength of the promoter increased the number of failures increased, further exemplifying the burden the lycopene pathway exerts on the recombinant host.

**Fig. 3:**
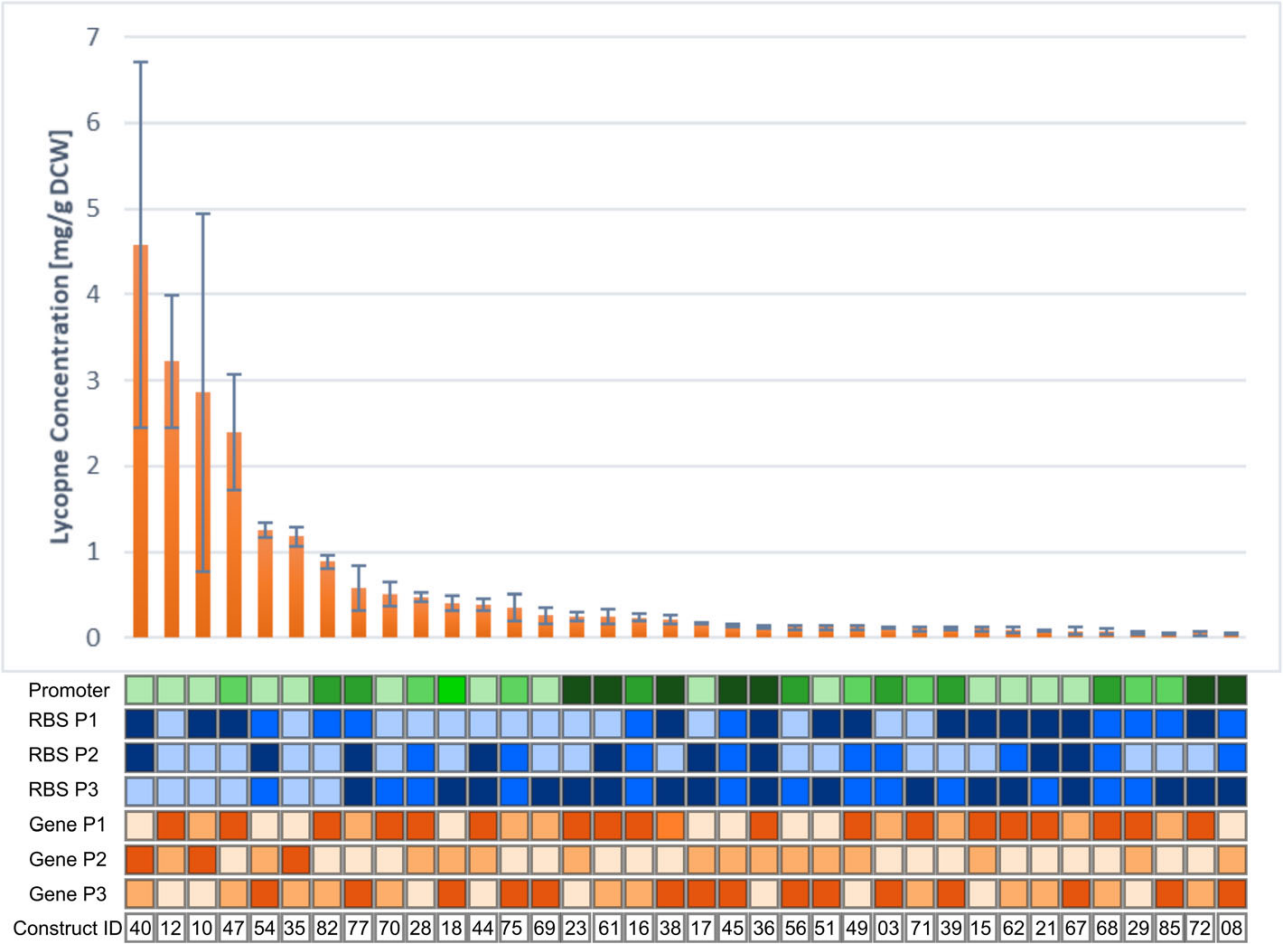
Lycopene production by different *E. coli* designs. 88 assemblies of the lycopene biosynthesis pathway from the design space were tested in *E. coli*. Of the 88 constructs, 46 produced colonies after transformation, of which 41 produced detectable lycopene content. Each clone was grown for 24 h in LB broth before lycopene content was determined as mg lycopene per dry cell weight (DCW). Error bars represent the standard deviation across three independent biological replicates

From the 41 constructs which produced lycopene, a range of relative concentrations were observed from a low of 0.05 mg/ g DCW to as high as 4.6 mg/ g DCW (Fig. 3). The measured lycopene titres for the 41 constructs were modelled using JMP. A least squares analysis was generated by JMP to estimate the maximum likelihood of a factor’s significance on production, known as the *p*-value. For the report, the LogWorth for each model factor defined as -log10(*p*-value) is utilised. This transformation adjusts *p*-values to provide an appropriate scale for graphing, a value that exceeds 2 is significant (reference JMP). The least squares fit model generated by the JMP model is summarised in the effect summary table ( Additional file 1: Table S2). The model ascertained that gene order or permutations (LogWorth value = 5.755) had the strongest significant negative effect on titres, followed by promoter strength (LogWorth value = 2.781).

It is not surprising that the promoter strength has an influence on lycopene concentration, it is well known that low expression rate is required so as not to divert vital metabolic flux away from the essential pathways which utilise IPP and/or FPP. This is the main reasoning behind promoters of medium to low strength being selected from SynBIS for use in the JMP data model. Operon organisation can also alter gene expression patterns, with higher expression for the gene farthest from the end of the operon (i.e. the more proximal gene) [36]. Therefore, the spatial organisation of an operon can help to modulate expression levels ensuring a balancing of the metabolic pathway. This can potentially improve production yields and minimise the burden imposed on the host.

The JMP software predicted that a design iteration of the weakest promoter, with weakest RBS in position 3 and with a gene order of [CrtE, CrtI, CrtB] or [CrtB, CrtI, CrtE] would have the highest probability of maximising titres. This demonstrated that the final enzyme of the pathway, CrtI, which converts phytoene to lycopene should be fixed in position 2, and that a low translation rate of enzyme in the position 3 (either CrtB or CrtI) is desirable. With this knowledge, any future designs can refer to the SynBIS data repository to find new parts which match the model parameters.

## Discussion

In this study an automated design-build workflow was implemented to assess a DoE approach for the synthesis of lycopene from a heterologous metabolic pathway in *E. coli*. Although the heterologous production of lycopene has previously been reported, what underlies this approach is utilisation of accurately characterised parts, where the part itself is associated to a part registry (database) with its characterisation data and metadata. The approach used in this paper is consistent with the core principles of synthetic biology. Which are based on the engineering principles of standardisation, characterisation and modularisation. So that, consistent with other areas of device development and manufacture, standard devices are built from standard components. In this case, the device being the lycopene biosynthetic pathway and the standard components being the parts.

Lycopene was chosen as an exemplar to illustrate implementation of an automated design and build workflow using a DNA toolkit. DNA toolkits are formed of standardised interchangeable biological parts, this means the design and build methodology from this paper can be applied to other heterologous biosynthetic operons. A flowchart outlining a step-by-step guide to the computer aided design of a biosynthetic pathway has been included within the supplementary information (Additional file 1: Figure S3).

In this paper, a multivariate approach for the construction of the lycopene operon resulted in a design space of 810 possible pathway configurations. The objective was to determine which configuration of components (parts) will give the largest lycopene output. The problem is that it can often be impractical to test each factorial change of design space to realise which part combinations is the optimal design. Consequently, researchers have found a Design of Experiment approach to examine a randomised multifactorial design space more beneficial. In this study the statistical model-based JMP software was as an experimental design and analysis tool.

To manage and connect the data flow between characterised parts of an assembly toolkit and DoE software an in-house software was developed (Additional file 1: Figure S1). This software limits the manual entering of variables, which can be laborious and prone to human error. Furthermore, the software ranks parts in order of strength. This is useful as the DoE software JMP can guide design specifications, helped by the ranking of parts. The utilisation of toolkits allows the determination of which strength parts to use with further designs. For instance, the model produced in this study inferred that product yields can be improved with weaker strength parts which direct transcription and translation. Any future designs can refer to SynBIS data repository to find new parts which match the model parameters. In contrast, the use of a randomised library of biological parts can result in a far more difficult and lengthy procedure to converge onto an optimal design.

The engineering principles of standardisation, characterisation and modularisation have encouraged the generation of DNA toolkits. DNA toolkits can be seen to promote the use of identical parts over bespoke designs. As DNA toolkits are formed of discrete and interchangeable biological parts, this in principle, enables predictive modelling and, therefore, does not require simultaneous optimisation of random combinatorial parts. Although, there are instances where a designed genetic regulatory network fails to perform as predicted when tested [37, 38]. The use of the DoE software JMP has the potential to observe interactions between parts and observe anomalous behaviour, particularly after multiple rounds of Design-Build-Test cycles. Nevertheless, researchers must consider the possible behaviour changes of individual parts with different metabolic pathway designs.

## Conclusion

For this study we have implemented a workflow to facilitate design automation and to pass data standards easily between different computational tools. In the study information from a set of standardised characterised parts was used to build a lycopene biosynthesis pathway with the aid of Design of Experiment software. We envisage the workflow utilised for this study will be very useful for the design and building of other metabolic pathway.

## Materials and methods

### Part preparation

A DNA toolkit of part plasmids containing promoters, CDS and terminator to build the lycopene biosynthesis pathway was prepared as follows; Parts were synthesised by commercial vendors (ATUM, USA; IDT, USA). DNA parts of less than 100 bp were ordered as 2 complementary single stranded oligonucleotides. The complementary oligonucleotides of equimolar concentration were annealed by heating to 96 °C for 5 min in a PCR machine before reducing the temperature to 23 °C at a ramp speed of 0.1 °C/sec. The final annealed product were ligated into a vector using CloneJET PCR Cloning Kit (Thermo Scientific,USA). DNA parts of greater then 100 bp were synthesised commercially into a self-replicating plasmid. The *E. coli* strain DH5α (NEB, USA) was used for all DNA cloning. Strains were maintained on Lysogeny broth (LB) or LB agar supplemented with ampicillin (50 μg mL^− 1^) for plasmid selection.

The part plasmids were isolated from *E. coli* using peqGOLD Plasmid Miniprep Kit I (PEQlab, Germany) according to the manufacturer’s protocols. The parts plasmids were assayed using the PicoGreen dsDNA reagent (ThermoFisher) to ensure each DNA part could be transferred successfully using acoustic liquid handling (Echo 550/525, Labcyte). DNA concentration of each part plasmids was quantified using a Nanodrop (ThermoFisher) and normalised to 76 nM with deionised water. A 1 mL aliquot of each normalised part plasmid were transferred to 96 deep-well plates for library storage.

To complete the DNA toolkit DNA linkers containing RBS parts were assembled. Phosphorylated oligonucleotides were synthesised with designs specifications corresponding to published protocol [34]. The oligonucleotides were annealed as above and normalised to concentration of 1 μM. 1 mL aliquot of normalised linker sequence was transferred to the same 96 deep-well plates for library storage.

### Building the lycopene metabolic pathway

The lycopene biosynthesis pathway was assembled using BASIC (Biopart Assembly Standard for Idempotent Cloning) [34]. The transfer of regents for the DNA assembly reaction was performed in an acoustic liquid handler (Echo 550, Labcyte) similarly to previously published method [35]. The enzymatic digest-ligation cycling reaction was performed in a PCR machine. The BASIC reaction was performed according to an existing protocol. The protocol management software AMOS [39] was used to coordinate the assembly by directing the acoustic dispensing of part assembly, linkers and assembly reaction components.

After the assembly a 1 μL aliquot of reaction mix was dispensed, using a CyBio FeliX, into a commercially bought chemical competent *E. coli* DH5α in a 96 well-plate format (NEB, USA). The 96 well-plate containing transformant mixture was placed in an ice-cold PCR Cooler (Eppendorf) before being transferred to a PCR machine set at 42 °C for a 30 s heat shock. After a one-hour recovery, six 3 μL aliquots (18 μL total) of the transformant mixture, from each well, was dispensed using a CyBio FeliX, onto LB agar in an Omnitray (ThermoFisher) supplemented with Kanamycin (35 μg mL^− 1^), with a four-minute drying time after each dispense. The plasmid isolation was performed according to an existing protocol utilising a PureLink Pro Quick96 Plasmid Purification Kit (ThermoFisher, Waltham, MA) [40]. The Fragment Analyzer dsDNA 930 (75 bp - 20,000 bp) and dsDNA 915 (35 bp - 50,000 bp) reagent kits (Advanced Analytical Technologies, Inc., Ames, IA, USA) were used to verify DNA constructs.

### Analysis and quantification of lycopene content of cells

*E. coli* colonies on Omnitray were picked using CyBio FeliX and cultured in 96-well deep volume plate containing 1 mL of LB media supplemented with kanamycin (35 μg mL^− 1^), plates were grown overnight at 37 °C with shaking at 600 rpm. An aliquot of 0.1 mL from the liquid culture was transferred to a 96-well plate and optical density at 600 nm was calculated, this was correlated to dry cell weight (DCW) with a ratio of DCW/OD = 0.36 and then corrected for full culture volume of *E. coli*. To obtain lycopene concentrations the bacterial cells from 96- deep well plate were pelleted by centrifuging at 4000 rpm for 10 min. The supernatant was removed, and bacterial cell pellet were washed in ddH_2_O. The bacterial cell pellet was resuspended in 1 ml acetone and incubated at 55 °C for 15 min to extract lycopene.

The supernatant was obtained by filtration through a 0.22 μm pore-size nylon membrane for LC-DAD analysis. Lycopene was detected and measured using an Agilent LC system with U*V*/Vis diode array detector. The LC column used was an Acquity UPLC Peptide BEH C18 column (2.1 × 100 mm, 1.7 μm, 300 Å, Waters). The mobile phases used were 1:1 (*v*/v) methanol in water (A) and 1:3 (v/v) of ethyl acetate in acetonitrile (B). Elution of the sample was carried out using the following gradient (t = time): t_0min_: 30% A; t_1min_ 0.1% A; t_6min_: 30% A; at a flow rate of 0.3 ml/min. The injection volume for the samples was 1 μl. Detection was performed at an absorbance of 450 nm and 471 nm simultaneously, with the peak area corresponding to each component integrated to provide a measure of abundance. Commercially available lycopene (Sigma-Aldrich) was dissolved in acetone as a standard and a standard curve was generated.

### Statistical analysis

A standard least squares regression model was produced using JMP Pro 13.2.0 (SAS Institute), with non-significant factors not being considered for future builds.

## Additional file

Additional file 1: The Supplementary Information. PDF file containing **Table S1** (part information of the lycopene biosynthetic pathway), **Table S2** (effect summary table), **Figure S1** (graphical illustration summary of SynBIS to DOE converter), **Figure S2** (E.coli colonies containing lycopene pathway), **Figure S3** (flowchart of the automated design) and **Figure S4** (a datasheet). (DOCX 2353 kb)

## Abbreviations

BASIC: Biopart Assembly Standard for Idempotent Cloning
CAD: Computer Aided Design
CDS: Coding Sequence
CSV: Comma Separated Variable
CrtB: phytoene synthase
CrtE: GGPP synthase
CrtI: phytoene desaturase
DCW: Dry Cell Weight
DICOM-SB: Digital Imaging and Communications in Medicine - Synthetic Biology
DoE: Design of Experiment
FPP: Farnesyl pyrophosphate
GFP: Green Fluorescent Protein
IPP: Isopentenyl pyrophosphate
LIMS: Laboratory Information Management System
MEP: Methylerythritol phosphate (non-Mevalonate pathway)
SBOL: Synthetic Biology Open Language
RBS: Ribosome Binding Site
RPU: Relative Promoter Unit
