# Supplementary material for: Utilising datasheets for the informed automated design and build of a synthetic metabolic pathway
Source: J Biol Eng. 2019 Jan 18;13:8. doi: 10.1186/s13036-019-0141-z (PMC6339355; doi:10.1186/s13036-019-0141-z)
Supplement: Supplementary file 1 — The Supplementary Information. PDF file containing Table S1 (part information of the lycopene biosynthetic pathway), Table S2 (effect summary table), Figure S1 (graphical illustration summary of SynBIS to DOE converter), Figure S2 (E.coli colonies containing lycopene pathway), Figure S3 (flowchart of the automated design) and Figure S4 (a datasheet). (DOCX 2353 kb) [file 13036_2019_141_MOESM1_ESM.docx]

**Table S1** the part composition and permutations of each lycopene biosynthetic pathway designed and constructed by AMOS. Each part is ranked according to the order as specified by JMP (SAS). The average lycopene concentration of each construct is shown where applicable. For constructs which failed to produce a colony an acronym of NC (No colony) is used. Where colonies grew but no lycopene was detected an acronym of ND (non-detectable) is used.

| **Design Iteration ID** | **Well position** | **Promoter** | | **RBS Position** | | | | | | **Gene Order** | | | **Lycopene Conc [mg/ g DCW]** |
| --- | --- | --- | --- | --- | --- | --- | --- | --- | --- | --- | --- | --- | --- |
|  |  |  |  | **First** | | **Second** | | **Third** | |  |  |  |  |
|  |  | **Name** | **Rank** | **Name** | **Rank** | **Name** | **Rank** | **Name** | **Rank** | **First** | **Second** | **Third** |  |
| 1 | A1 | J23106 | 2 | BBa_B0064 | 2 | BBa_B0064 | 2 | BASIC L1 | 1 | CRTI | CRTB | CRTE | NC |
| 2 | B1 | J23101 | 5 | BBa_B0034 | 3 | BBa_B0034 | 3 | BASIC L1 | 1 | CRTB | CRTE | CRTI | NC |
| 3 | C1 | J23106 | 2 | BASIC L1 | 1 | BBa_B0064 | 2 | BBa_B0064 | 2 | CRTB | CRTE | CRTI | 0.007781 |
| 4 | D1 | J23106 | 2 | BASIC L1 | 1 | BBa_B0064 | 2 | BBa_B0064 | 2 | CRTE | CRTI | CRTB | NC |
| 5 | E1 | apFAB53 | 3 | BBa_B0034 | 3 | BBa_B0034 | 3 | BBa_B0034 | 3 | CRTB | CRTI | CRTE | NC |
| 6 | F1 | Bba_K137085 | 1 | BBa_B0064 | 2 | BASIC L1 | 1 | BASIC L1 | 1 | CRTE | CRTB | CRTI | NC |
| 7 | G1 | apFAB53 | 3 | BBa_B0064 | 2 | BASIC L1 | 1 | BBa_B0064 | 2 | CRTE | CRTI | CRTB | NC |
| 8 | H1 | J23101 | 5 | BBa_B0064 | 2 | BBa_B0064 | 2 | BBa_B0034 | 3 | CRTE | CRTB | CRTI | 0.015288 |
| 9 | H11 | J23101 | 5 | BASIC L1 | 1 | BBa_B0034 | 3 | BASIC L1 | 1 | CRTI | CRTB | CRTE | NC |
| 10 | A2 | Bba_K137085 | 1 | BBa_B0034 | 3 | BASIC L1 | 1 | BASIC L1 | 1 | CRTB | CRTI | CRTE | 2.086665 |
| 11 | A3 | J23101 | 5 | BBa_B0034 | 3 | BBa_B0034 | 3 | BBa_B0034 | 3 | CRTE | CRTI | CRTB | 0.76285 |
| 12 | A4 | Bba_K137085 | 1 | BASIC L1 | 1 | BASIC L1 | 1 | BASIC L1 | 1 | CRTI | CRTB | CRTE | NC |
| 13 | A5 | apFAB53 | 3 | BBa_B0064 | 2 | BBa_B0034 | 3 | BASIC L1 | 1 | CRTB | CRTE | CRTI | NC |
| 14 | A6 | J23101 | 5 | BASIC L1 | 1 | BBa_B0034 | 3 | BBa_B0034 | 3 | CRTB | CRTE | CRTI | 0.019657 |
| 15 | A7 | Bba_K137085 | 1 | BBa_B0034 | 3 | BASIC L1 | 1 | BBa_B0034 | 3 | CRTI | CRTB | CRTE | 0.031749 |
| 16 | A8 | J23106 | 2 | BBa_B0064 | 2 | BBa_B0064 | 2 | BBa_B0064 | 2 | CRTI | CRTE | CRTB | 0.039454 |
| 17 | A9 | Bba_K137085 | 1 | BASIC L1 | 1 | BBa_B0034 | 3 | BBa_B0034 | 3 | CRTE | CRTB | CRTI | 0.002229 |
| 18 | A10 | J23108 | 4 | BASIC L1 | 1 | BASIC L1 | 1 | BBa_B0034 | 3 | CRTE | CRTB | CRTI | 0.082983 |
| 19 | A11 | J23106 | 2 | BBa_B0064 | 2 | BBa_B0064 | 2 | BASIC L1 | 1 | CRTB | CRTE | CRTI | NC |
| 20 | B2 | Bba_K137085 | 1 | BASIC L1 | 1 | BASIC L1 | 1 | BBa_B0034 | 3 | CRTB | CRTI | CRTE | NC |
| 21 | B3 | Bba_K137085 | 1 | BBa_B0034 | 3 | BBa_B0034 | 3 | BBa_B0064 | 2 | CRTI | CRTE | CRTB | 0.009797 |
| 22 | B4 | J23106 | 2 | BBa_B0034 | 3 | BBa_B0064 | 2 | BBa_B0064 | 2 | CRTE | CRTI | CRTB | NC |
| 23 | B5 | J23101 | 5 | BASIC L1 | 1 | BASIC L1 | 1 | BBa_B0034 | 3 | CRTI | CRTB | CRTE | 0.052992 |
| 24 | B6 | Bba_K137085 | 1 | BBa_B0034 | 3 | BBa_B0034 | 3 | BASIC L1 | 1 | CRTI | CRTB | CRTE | NC |
| 25 | B7 | Bba_K137085 | 1 | BBa_B0034 | 3 | BASIC L1 | 1 | BASIC L1 | 1 | CRTB | CRTE | CRTI | NC |
| 26 | B8 | apFAB53 | 3 | BBa_B0064 | 2 | BASIC L1 | 1 | BBa_B0064 | 2 | CRTB | CRTI | CRTE | NC |
| 27 | B9 | J23106 | 2 | BASIC L1 | 1 | BBa_B0034 | 3 | BBa_B0034 | 3 | CRTB | CRTI | CRTE | NC |
| 28 | B10 | apFAB53 | 3 | BASIC L1 | 1 | BBa_B0064 | 2 | BBa_B0064 | 2 | CRTI | CRTB | CRTE | 0.048636 |
| 29 | B11 | apFAB53 | 3 | BBa_B0064 | 2 | BASIC L1 | 1 | BBa_B0064 | 2 | CRTI | CRTB | CRTE | 0.020872 |
| 30 | C2 | apFAB53 | 3 | BASIC L1 | 1 | BBa_B0064 | 2 | BBa_B0064 | 2 | CRTB | CRTI | CRTE | NC |
| 31 | C3 | J23101 | 5 | BBa_B0034 | 3 | BBa_B0034 | 3 | BBa_B0064 | 2 | CRTB | CRTI | CRTE | NC |
| 32 | C4 | Bba_K137085 | 1 | BASIC L1 | 1 | BBa_B0034 | 3 | BBa_B0034 | 3 | CRTE | CRTI | CRTB | NC |
| 33 | C5 | J23106 | 2 | BBa_B0064 | 2 | BASIC L1 | 1 | BBa_B0064 | 2 | CRTE | CRTI | CRTB | NC |
| 34 | C6 | J23101 | 5 | BASIC L1 | 1 | BBa_B0034 | 3 | BASIC L1 | 1 | CRTE | CRTI | CRTB | NC |
| 35 | C7 | Bba_K137085 | 1 | BASIC L1 | 1 | BASIC L1 | 1 | BASIC L1 | 1 | CRTE | CRTI | CRTB | 0.112076 |
| 36 | C8 | J23101 | 5 | BBa_B0034 | 3 | BBa_B0034 | 3 | BBa_B0034 | 3 | CRTI | CRTB | CRTE | 0.014143 |
| 37 | C9 | J23106 | 2 | BBa_B0034 | 3 | BBa_B0064 | 2 | BASIC L1 | 1 | CRTB | CRTI | CRTE | NC |
| 38 | C10 | J23101 | 5 | BBa_B0034 | 3 | BASIC L1 | 1 | BBa_B0034 | 3 | CRTB | CRTE | CRTI | 0.045552 |
| 39 | C11 | J23106 | 2 | BBa_B0034 | 3 | BASIC L1 | 1 | BBa_B0064 | 2 | CRTB | CRTE | CRTI | 0.021248 |
| 40 | D2 | Bba_K137085 | 1 | BBa_B0034 | 3 | BBa_B0034 | 3 | BASIC L1 | 1 | CRTE | CRTI | CRTB | 2.132293 |
| 41 | D3 | apFAB53 | 3 | BBa_B0064 | 2 | BBa_B0034 | 3 | BBa_B0034 | 3 | CRTI | CRTE | CRTB | ND |
| 42 | D4 | apFAB53 | 3 | BASIC L1 | 1 | BBa_B0064 | 2 | BASIC L1 | 1 | CRTE | CRTB | CRTI | NC |
| 43 | D5 | J23108 | 4 | BASIC L1 | 1 | BBa_B0034 | 3 | BASIC L1 | 1 | CRTB | CRTI | CRTE | NC |
| 44 | D6 | Bba_K137085 | 1 | BASIC L1 | 1 | BBa_B0034 | 3 | BBa_B0034 | 3 | CRTI | CRTB | CRTE | 0.068447 |
| 45 | D7 | J23101 | 5 | BBa_B0064 | 2 | BBa_B0064 | 2 | BBa_B0064 | 2 | CRTE | CRTB | CRTI | 0.022534 |
| 46 | D8 | J23106 | 2 | BBa_B0034 | 3 | BASIC L1 | 1 | BBa_B0034 | 3 | CRTB | CRTI | CRTE | NC |
| 47 | D9 | apFAB53 | 3 | BBa_B0034 | 3 | BASIC L1 | 1 | BASIC L1 | 1 | CRTI | CRTE | CRTB | 0.675336 |
| 48 | D10 | J23101 | 5 | BBa_B0064 | 2 | BBa_B0064 | 2 | BBa_B0064 | 2 | CRTB | CRTI | CRTE | NC |
| 49 | D11 | apFAB53 | 3 | BBa_B0034 | 3 | BBa_B0064 | 2 | BBa_B0064 | 2 | CRTI | CRTB | CRTE | 0.026735 |
| 50 | E2 | Bba_K137085 | 1 | BBa_B0064 | 2 | BBa_B0064 | 2 | BBa_B0064 | 2 | CRTB | CRTI | CRTE | NC |
| 51 | E3 | Bba_K137085 | 1 | BBa_B0034 | 3 | BASIC L1 | 1 | BBa_B0034 | 3 | CRTE | CRTB | CRTI | 0.027293 |
| 52 | E4 | J23108 | 4 | BBa_B0064 | 2 | BBa_B0034 | 3 | BBa_B0064 | 2 | CRTE | CRTI | CRTB | NC |
| 53 | E5 | J23101 | 5 | BBa_B0034 | 3 | BASIC L1 | 1 | BASIC L1 | 1 | CRTE | CRTB | CRTI | NC |
| 54 | E6 | Bba_K137085 | 1 | BBa_B0064 | 2 | BBa_B0034 | 3 | BBa_B0064 | 2 | CRTE | CRTB | CRTI | 0.09035 |
| 55 | E7 | J23101 | 5 | BASIC L1 | 1 | BASIC L1 | 1 | BASIC L1 | 1 | CRTB | CRTE | CRTI | NC |
| 56 | E8 | J23106 | 2 | BASIC L1 | 1 | BASIC L1 | 1 | BBa_B0064 | 2 | CRTE | CRTB | CRTI | 0.02419 |
| 57 | E9 | J23101 | 5 | BBa_B0064 | 2 | BBa_B0064 | 2 | BBa_B0034 | 3 | CRTB | CRTI | CRTE | NC |
| 58 | E10 | J23106 | 2 | BBa_B0064 | 2 | BBa_B0034 | 3 | BBa_B0064 | 2 | CRTI | CRTB | CRTE | 0.032705 |
| 59 | E11 | apFAB53 | 3 | BBa_B0064 | 2 | BBa_B0064 | 2 | BBa_B0034 | 3 | CRTE | CRTI | CRTB | NC |
| 60 | F2 | Bba_K137085 | 1 | BASIC L1 | 1 | BBa_B0034 | 3 | BASIC L1 | 1 | CRTB | CRTE | CRTI | NC |
| 61 | F3 | J23101 | 5 | BASIC L1 | 1 | BBa_B0034 | 3 | BBa_B0034 | 3 | CRTI | CRTE | CRTB | 0.084753 |
| 62 | F4 | Bba_K137085 | 1 | BBa_B0034 | 3 | BBa_B0064 | 2 | BBa_B0034 | 3 | CRTI | CRTE | CRTB | 0.041466 |
| 63 | F5 | J23101 | 5 | BASIC L1 | 1 | BASIC L1 | 1 | BBa_B0034 | 3 | CRTE | CRTI | CRTB | NC |
| 64 | F6 | Bba_K137085 | 1 | BBa_B0034 | 3 | BASIC L1 | 1 | BBa_B0034 | 3 | CRTE | CRTI | CRTB | NC |
| 65 | F7 | J23101 | 5 | BASIC L1 | 1 | BBa_B0034 | 3 | BASIC L1 | 1 | CRTE | CRTB | CRTI | NC |
| 66 | F8 | J23106 | 2 | BBa_B0034 | 3 | BBa_B0034 | 3 | BBa_B0034 | 3 | CRTE | CRTB | CRTI | 0.006588 |
| 67 | F9 | Bba_K137085 | 1 | BBa_B0034 | 3 | BBa_B0034 | 3 | BBa_B0034 | 3 | CRTB | CRTE | CRTI | 0.037226 |
| 68 | F10 | J23106 | 2 | BBa_B0064 | 2 | BBa_B0064 | 2 | BBa_B0064 | 2 | CRTI | CRTE | CRTB | 0.036849 |
| 69 | F11 | Bba_K137085 | 1 | BASIC L1 | 1 | BASIC L1 | 1 | BBa_B0034 | 3 | CRTB | CRTE | CRTI | 0.097896 |
| 70 | G2 | Bba_K137085 | 1 | BASIC L1 | 1 | BASIC L1 | 1 | BBa_B0064 | 2 | CRTI | CRTE | CRTB | 0.143761 |
| 71 | G3 | apFAB53 | 3 | BASIC L1 | 1 | BASIC L1 | 1 | BBa_B0034 | 3 | CRTI | CRTE | CRTB | 0.025918 |
| 72 | G4 | J23101 | 5 | BBa_B0034 | 3 | BASIC L1 | 1 | BBa_B0034 | 3 | CRTI | CRTE | CRTB | 0.023519 |
| 73 | G5 | apFAB53 | 3 | BBa_B0034 | 3 | BBa_B0064 | 2 | BBa_B0064 | 2 | CRTE | CRTB | CRTI | NC |
| 74 | G6 | J23101 | 5 | BBa_B0034 | 3 | BASIC L1 | 1 | BASIC L1 | 1 | CRTI | CRTB | CRTE | ND |
| 75 | G7 | apFAB53 | 3 | BASIC L1 | 1 | BBa_B0064 | 2 | BBa_B0064 | 2 | CRTB | CRTE | CRTI | 0.158291 |
| 76 | G8 | J23101 | 5 | BBa_B0034 | 3 | BASIC L1 | 1 | BASIC L1 | 1 | CRTE | CRTI | CRTB | NC |
| 77 | G9 | J23106 | 2 | BBa_B0064 | 2 | BBa_B0034 | 3 | BBa_B0034 | 3 | CRTB | CRTE | CRTI | 0.263819 |
| 78 | G10 | Bba_K137085 | 1 | BASIC L1 | 1 | BBa_B0034 | 3 | BASIC L1 | 1 | CRTI | CRTE | CRTB | NC |
| 79 | G11 | Bba_K137085 | 1 | BBa_B0064 | 2 | BBa_B0034 | 3 | BASIC L1 | 1 | CRTB | CRTI | CRTE | NC |
| 80 | H2 | apFAB53 | 3 | BBa_B0034 | 3 | BBa_B0064 | 2 | BBa_B0064 | 2 | CRTB | CRTE | CRTI | ND |
| 81 | H3 | J23101 | 5 | BASIC L1 | 1 | BASIC L1 | 1 | BASIC L1 | 1 | CRTB | CRTI | CRTE | NC |
| 82 | H4 | J23106 | 2 | BBa_B0064 | 2 | BASIC L1 | 1 | BASIC L1 | 1 | CRTI | CRTE | CRTB | 0.0765 |
| 83 | H5 | J23101 | 5 | BASIC L1 | 1 | BBa_B0064 | 2 | BASIC L1 | 1 | CRTI | CRTE | CRTB | NC |
| 84 | H6 | apFAB53 | 3 | BBa_B0064 | 2 | BBa_B0064 | 2 | BASIC L1 | 1 | CRTE | CRTI | CRTB | NC |
| 85 | H7 | apFAB53 | 3 | BBa_B0064 | 2 | BASIC L1 | 1 | BBa_B0034 | 3 | CRTB | CRTE | CRTI | 0.004168 |
| 86 | H8 | J23106 | 2 | BBa_B0064 | 2 | BBa_B0064 | 2 | BBa_B0034 | 3 | CRTI | CRTB | CRTE | 0.202234 |
| 87 | H9 | J23101 | 5 | BBa_B0034 | 3 | BBa_B0034 | 3 | BASIC L1 | 1 | CRTI | CRTE | CRTB | NC |
| 88 | H10 | J23108 | 4 | BBa_B0034 | 3 | BBa_B0034 | 3 | BASIC L1 | 1 | CRTE | CRTB | CRTI | NC |

Table S2 The fit least square model generated by the JMP model is summarised in the effect summary table


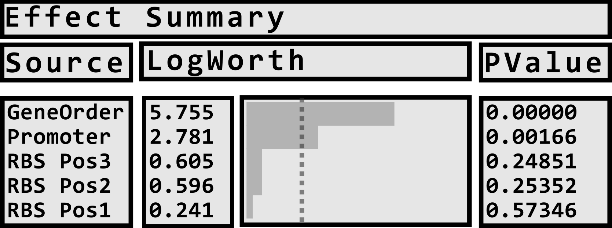


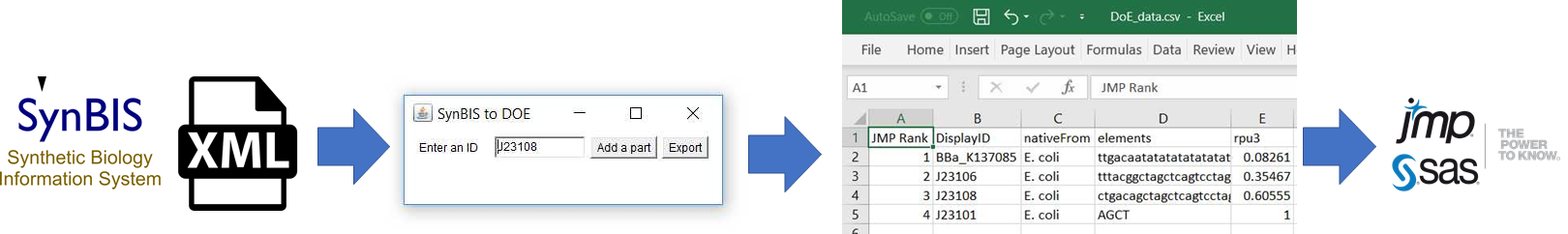


Figure S1 shows the graphical illustration summary of SynBIS to DOE converter used to facilitate ranking and entry of parts in JMP. A Graphic user interface allows users to define the part required by entering the ID. This ID is set within the SynBIS XML framework. By clicking ‘Add a part’ further parts can be added. Once user clicks ‘export’ part information is uploaded to a CSV file. The software ranks the parts, in this case constitutive promoters are ranked based on strength. The CSV file can be uploaded to JMP and parts defined as discrete numeric values with the number related to the rank order


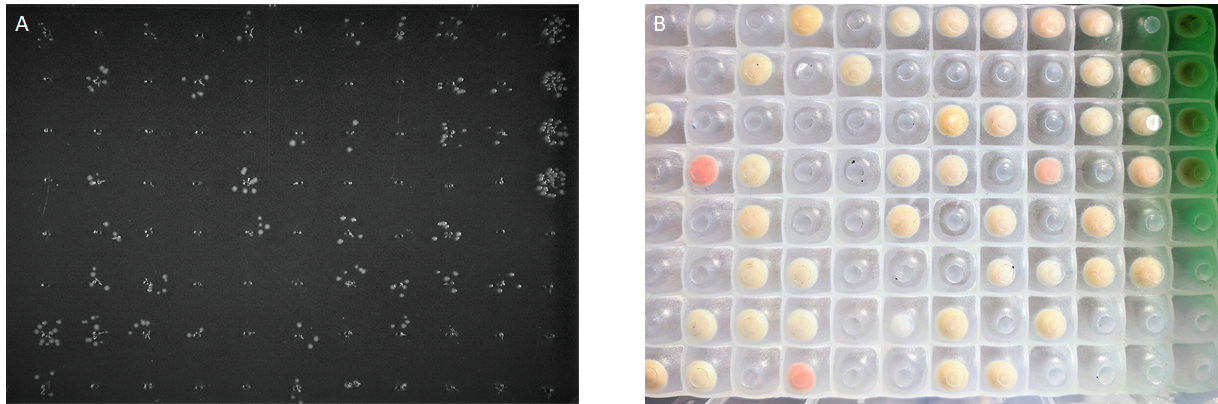


Figure S2 (A) Each lycopene biosynthetic pathway from Table S1 were transformed into *E. coli* and grown on LB agar supplemented with Kanamycin, the design iteration ID and corresponding plate position are shown in Table S1. (B) After 24 hours growth on LB agar colonies were placed into liquid culture within a 96 deep well plate and grown overnight to determine the lycopene concentrations.


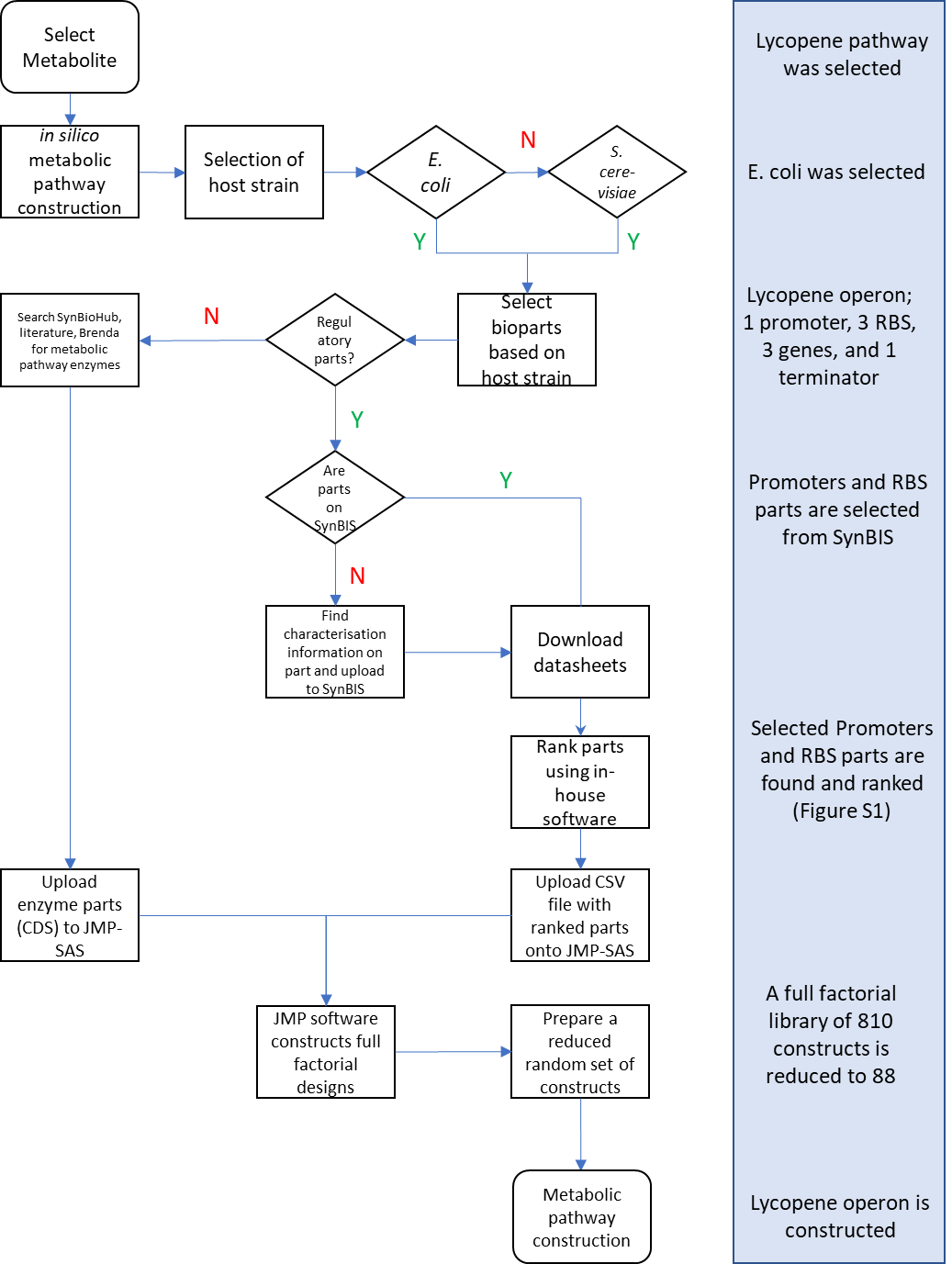
Figure S3 Flowchart representing the automated design of a biosynthetic pathway


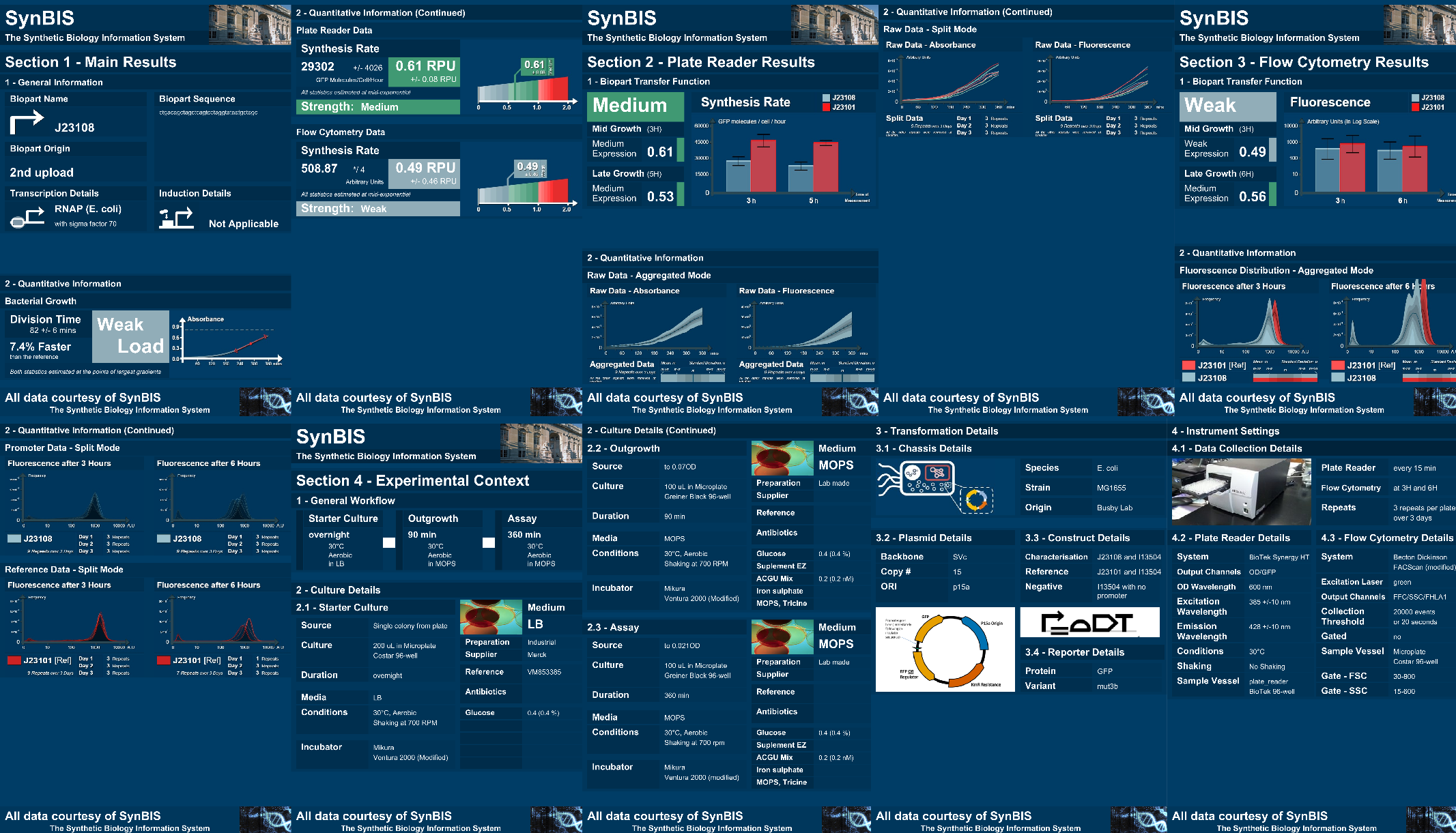


Figure S4 A screenshot of a datasheet from the constitutive promoter part J23108 from the SynBIS website.
